# Does MRI-Detected Cranial Nerve Involvement Affect the Prognosis of Locally Advanced Nasopharyngeal Carcinoma Treated with Intensity Modulated Radiotherapy?

**DOI:** 10.1371/journal.pone.0100571

**Published:** 2014-06-25

**Authors:** Jingfeng Zong, Shaojun Lin, Yunbin Chen, Bingyi Wang, Youping Xiao, Jin Lin, Rui Li, Jianji Pan

**Affiliations:** 1 Provincial Clinical College, Fujian Medical University, Fuzhou City, Fujian Province, People's Republic of China; 2 Department of Radiation Oncology, Fujian Provincial Cancer Hospital, Fuzhou City, Fujian Province, People's Republic of China; 3 Fujian Provincial Key Laboratory of Translational Cancer Medicine, Fujian Provincial Cancer Hospital, Fuzhou City, Fujian Province, People's Republic of China; 4 Department of Radiology, Fujian Provincial Cancer Hospital, Fuzhou City, Fujian Province, People's Republic of China; The George Washington University, United States of America

## Abstract

Nasopharyngeal carcinoma (NPC) is one of the common cancers in South China. It can easily invade into cranial nerves, especially in patients with local advanced disease. Despite the fact that the magnetic resonance imaging (MRI) findings are not always consistent with the symptoms of CN palsy, MRI is recommended for the detection of CN involvement (CNI). However, the prognostic impact of MRI-detected CNI in NPC patients is still controversial. To investigate the prognostic value of MRI detected CNI, we performed a retrospective analysis on the clinical data of 375 patients with NPC who were initially diagnosed by MRI. All patients had T3-4 disease and received radical intensity modulated radiation therapy (IMRT) as their primary treatment. The incidence of MRI-detected CNI was 60.8%. A higher incidence of MRI-detected CNI was observed in T4 disease compared with T3 disease (96.8% vs. 42.8%, P<0.001), and a higher incidence was also found in patients with Stage IV disease compared with those with Stage III disease (91.5% vs. 42.3%; P<0.001). The local relapse-free survival (LRFS), distant metastasis-free survival (DMFS), and overall survival (OS) of patients with T3 disease, with or without MRI-detected CNI, was superior to that of patients with T4 disease (P<0.05). No significant differences in LRFS, DMFS or OS were observed between T3 patients with or without MRI-detected CNI. The survival of Stage III patients with or without MRI-detected CNI was significantly superior to that of Stage IV patients (P<0.01), but there was no significant difference between Stage III patients with or without MRI-detected CNI for all endpoints. Therefore, when treated with IMRT, MRI-detected CNI in patients with NPC does not appear to affect the prognosis. In patients with clinical T3 disease, the presence of MRI-detected CNI is not sufficient evidence for defining T4 disease.

## Introduction

Nasopharyngeal carcinoma (NPC) is one of the common cancers in South China. NPC can easily infiltrate into the surrounding structures, such as parapharyngeal space, cavernous sinus and the cranial nerve (CN) [1]–[3]. The reported incidence of CN palsy in NPC is about 8.9%–10.4% [4]–[6]. Upon development of CN palsy, the prognosis is poor and these patients are staged as T4 disease according to the 7th edition American Joint Committee on Cancer (AJCC) TNM staging system [7], [8].

Recently, some researchers reported that Magnetic Resonance Imaging detected (MRI-detected) CN involvement (CNI) in NPC patients, with or without CN palsy, had a poor prognosis and proposed taking MRI-detected CNI into account in a future staging system [4]. Once the proposal is confirmed, it would modify the current staging criteria and affect the clinical treatment strategy, especially in Stage T3 patients with MRI-detected CNI.

With the extensive use of intensity modulated radiation therapy (IMRT), the local control rate has exceeded 90%, even in locally advanced NPC [9]–[11]. However, the prognosis of MRI-detected CNI NPC treated with IMRT has rarely been previously reported. In the present study, the clinical data of 375 local advanced NPC patients treated with IMRT were collected and retrospectively reviewed, the outcomes were evaluated and the prognosis of patients with MRI-detected CNI were analyzed to provide reference for a future staging system revision.

## Materials and Methods

### Patient characteristics

We collected the data of 375 newly diagnosed NPC patients without distant organ metastasis, who were classified as Stage T3/T4 according to the 7th edition AJCC TNM staging system and treated at Fujian Provincial Cancer Hospital between June 1, 2005 and July 31, 2009. The retrospective analysis was approved by Fujian Provincial Cancer Hospital Institutional Review Board. Although consent was not specifically obtained for this retrospective review, all information had been anonymized and de-identified prior to its analysis. The clinical characteristics of patients are summarized in Table 1. The patients included 297 men and 78 women (male: female ratio, 3.8∶1), with a median age of 44 years (range, 10–77 years). The majority of the patients (95.4%; 358/375) had World Health Organization (WHO) type III histopathology, 10 patients (2.7%) with WHO type II, and 7 patients (1.9%) with WHO type I.

**Table 1:** Clinical characteristics of patients.

Characteristics	Patients (%)
Age (years)	
<50	231 (61.6)
≥50	144 (38.4)
Gender	
Male	297 (79.2)
Female	78 (20.8)
Histology	
WHO grade I	7 (1.9)
WHO grade II	10 (2.7)
WHO grade III	358 (95.4)
T-stage	
T3	250 (66.7)
T4	125 (33.3)
N-stage	
N0	48 (12.8)
N1	198 (52.8)
N2	99 (26.4)
N3	30 (8.0)
Clinical stage	
Stage III	234 (62.4)
Stage IV	141 (37.6)
Treatment	
RT alone	30(8)
ICT-RT	109 (29.1)
ICT-RT-ACT	89 (23.7)
ICT-CCRT	72 (19.2)
ICT-CCRT-ACT	52 (13.9)
CCRT	11 (2.9)
CCRT-ACT	7 (1.9)
RT-ACT	5 (1.3)

*Abbreviations*: WHO = World Health Organization; RT =  radiotherapy; ICT =  Induction chemotherapy.

CCRT =  concurrent chemoradiotherapy, ACT = adjuvant chemotherapy.

### Pretreatment workup and diagnosis of MRI-detected CN involvement

Pretreatment work-up included a complete history and physical examination, standard laboratory tests, nasopharynx fiberoptic examination, head and neck MRI scan according to the protocol [12], chest radiograph or CT scan, abdominal ultrasound, bone scan, and dental evaluation.

**Figure 1:**
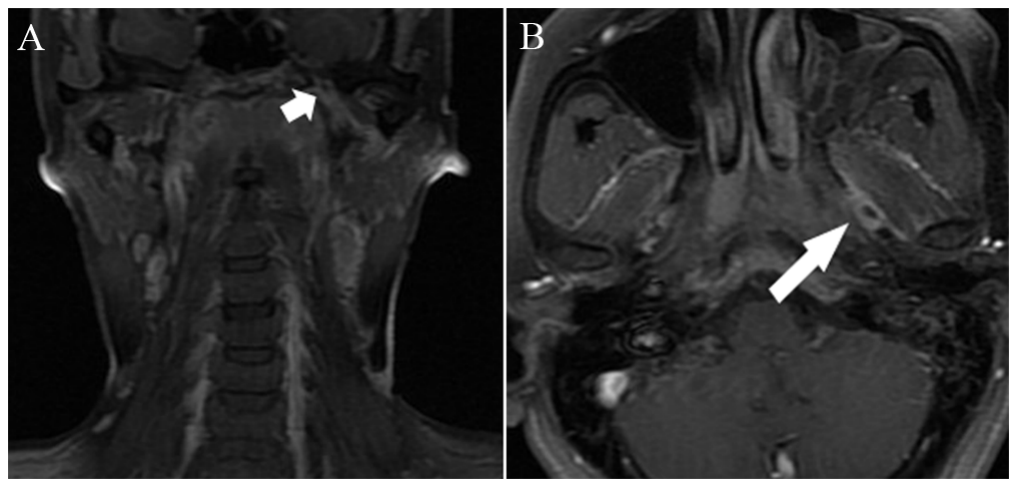
39-year-old woman with nasopharyngeal carcinoma with cranial nerve invasion: (A) enhanced coronal T1-weighted image with fat suppression shows the enlarged left foramen ovale with abnormal enhancement (short arrow); (B) the enhanced axial T1-weighted image with fat suppression shows the enlargement and abnormal enhancement of cranial nerve V3 (long arrow).

**Figure 2:**
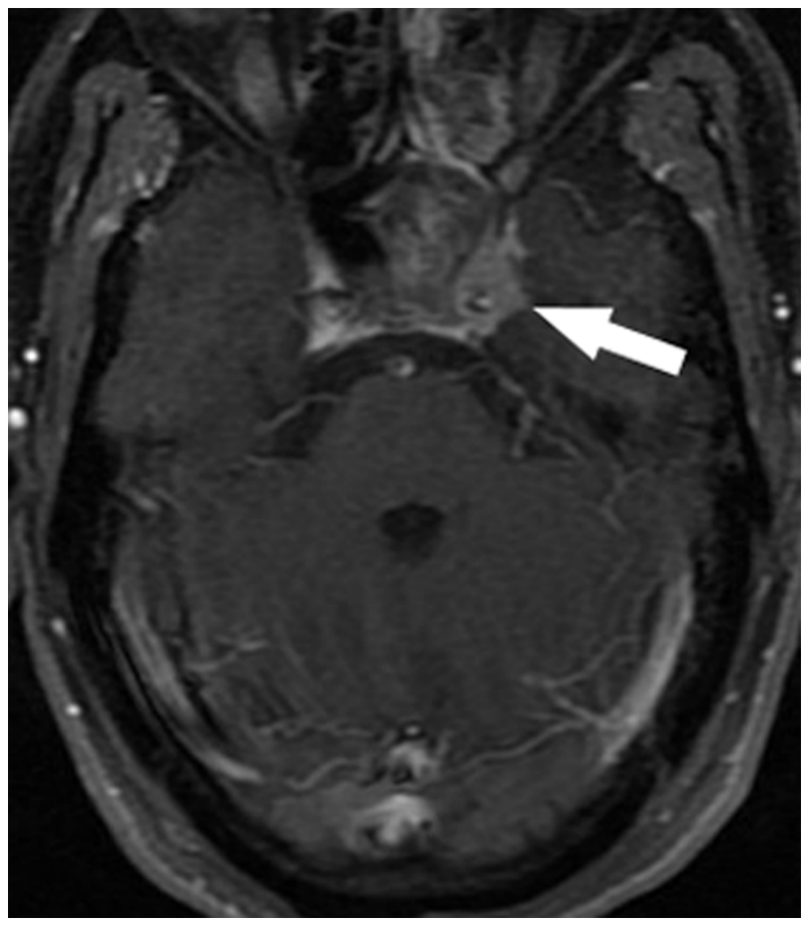
42-year-old man with nasopharyngeal carcinoma with cranial nerve invasion: enhanced axial T1-weighted image with fat suppression shows tumor invasion in the left cavernous sinus (arrow).

**Figure 3:**
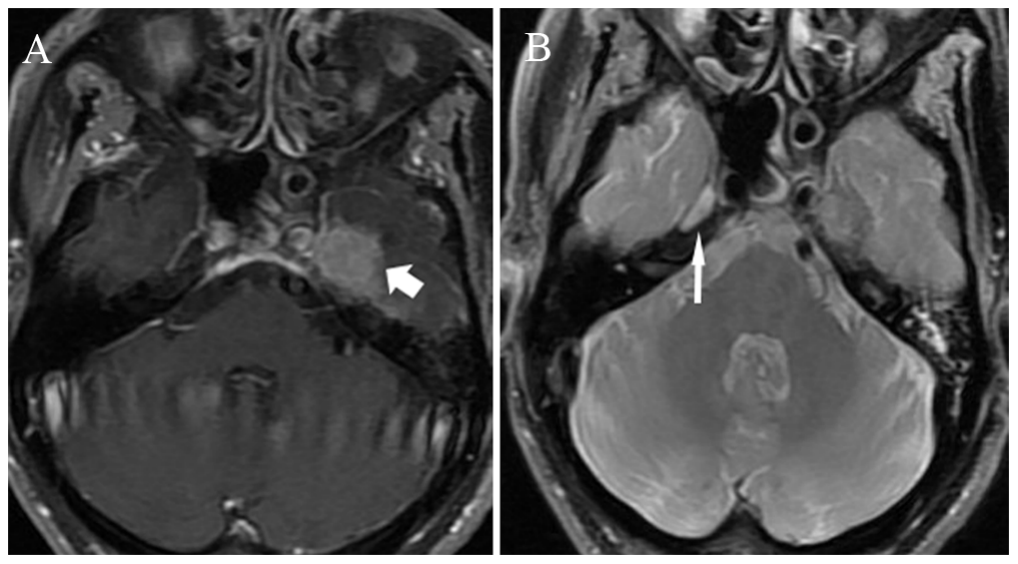
72-year-old man with nasopharyngeal carcinoma with cranial nerve invasion: (A) enhanced axial T1-weighted image with fat suppression shows the effacement of the left gasserian ganglion (short arrow); (B) axial PD weighted imaging shows the normal right gasserian ganglion (long arrow).

The diagnostic criteria for MRI-detected CN involvement were as follows [13]–[15]: i) abnormal CN thickening with enhancement after intravenous administration of contrast material; ii) cavernous sinus expansion and abnormal enhancement after enhanced scanning; iii) asymmetrical effacement of the Meckel's cave or gasserian ganglion by enhancing soft tissue; iv) widening and/or destruction of and/or excessive enhancement within neural foramina (Figs. 1, 2, 3).

### Treatment

All patients were initially treated with definitive IMRT. A detailed description of the IMRT had been published previously [11]. A total of 345 patients (92%) received platinum-based chemotherapy: the sequence used was induction in 29.1%, concurrent 2.9%, adjuvant 1.3%, concurrent-adjuvant 1.9%, induction-concurrent 19.2%, induction-adjuvant 23.7%, and induction-concurrent-adjuvant 13.9%. Whenever possible, patients who developed relapse or persistent tumor after completing initial treatment received salvage treatments (including intracavitary brachytherapy, surgery, or adjuvant chemotherapy).

### Follow up and statistical analysis

The median follow-up time was 57 months (range, 6–96 months). Overall survival (OS) was calculated from the first day of diagnosis to the date of death or the last follow-up. Local relapse-free survival (LRFS) was calculated from the first day of diagnosis to the date of local relapse, and the distant metastasis-free survival (DMFS) from the first day of diagnosis to the date of distant metastasis. The outcomes were evaluated in August, 2013.

The survival data were analyzed with SPSS software, version 18.0 (SPSS, Inc., Chicago, IL, USA). The MRI-detected CNI incidence in different T and N classifications were analyzed and compared with the chi-square test. Survival curves were created with the Kaplan-Meier method and compared with the log-rank test. Two-tailed Ρ values <0.05 were considered statistically significant.

## Results

### Failure patterns

At the last following up, 94 (25.1%) patients had succumbed to their disease and 281 (74.9%) remained alive. The 5-year OS, DMFS and LRFS were 74.2%, 79.4% and 94.3% respectively. Local recurrences, regional recurrences and distant metastases developed in 17 (4.5%), 3 (0.8%) and 70 (18.7%) patients, whereas 7 (1.9%) patients presented with both locoregional recurrences and distant metastases.

### Incidence and prognosis of MRI-detected CN involvement

A total of 228 (60.8%) patients were diagnosed with MRI-detected CNI. The CN V involvement was the most frequent followed by CN III, IV, and XII, sequentially. Only 41 (18%) of MRI-detected CNI patients presented with CN palsy. However, there were a total of five cases of CN palsy that we failed to discover by MRI in four patients, including one patient with CN VII, one patient with CN XI and XII, and two patients with CN V.

The incidence of MRI-detected CNI in different T and N classifications are summarized in Table 2. A higher incidence of MRI-detected CNI was observed in Stage T4 compared to Stage T3 (96.8% vs. 42.8%; P<0.001), as well as in Stage IV compared to Stage III (91.5% vs. 42.3%; P<0.001). These differences were statistically significant. While the difference between different N stages (N0-1 to N2 -3) was not significant (59.8% vs.62.8%, P = 0.567).

**Table 2:** Incidence and survival in patients with and without MRI-detected CNI.

	with MRI-detected CNI		Without MRI-detected CNI	P Value
T-stage (%)				<0.001
T3		107 (42.8)	143 (57.2)	
T4		121 (96.8)	4 (3.2)	
N-stage (%)				0.567
N0-1		147(59.8)	99 (40.2)	
N2-3		81 (62.8)	48 (37.2)	
Clinical stage (%)				<0.001
Stage III		99 (42.3)	135 (57.7)	
Stage IV		129 (91.5)	12 (8.5)	
5-year Survival (%)				
LRFS		90.2	99.2	0.001(0.102*)
DMFS		77.1	81.2	0.402
OS		71.9	77.7	0.134

Abbreviations: MRI  =  magnetic resonance imaging; CNI  =  cranial nerve involvement; LRFS  =  local relapse-free survival; DMFS  =  distant metastasis-free survival; OS =  overall survival.

*P Value was calculated using Cox proportional hazards model after adjusted for T and N classification.

The 5-year DMFS or OS of patients with or without MRI-detected CNI were not significantly different (77.1% vs. 81.2%, χ^2^ = 0.702, P = 0.402; 71.9% vs. 77.7%, χ^2^ = 2.242, P = 0.134). Although the difference in LRFS for patients with or without MRI-detected CNI was significant (90.2% vs. 99.2%, χ^2^ = 11.119, P = 0.001), it lost statistical significance after adjusting for T and N classification (P = 0.102) (Table 2).

### MRI-detected CN involvement impact in the current AJCC staging system

To evaluate the impact of MRI-detected CNI in the current AJCC staging system, the Stage T3 and Stage III patients were classified into different categories by MRI-detected CNI: Stage T3 with and without MRI-detected CNI, Stage III with and without MRI-detected CNI.

**Figure 4:**
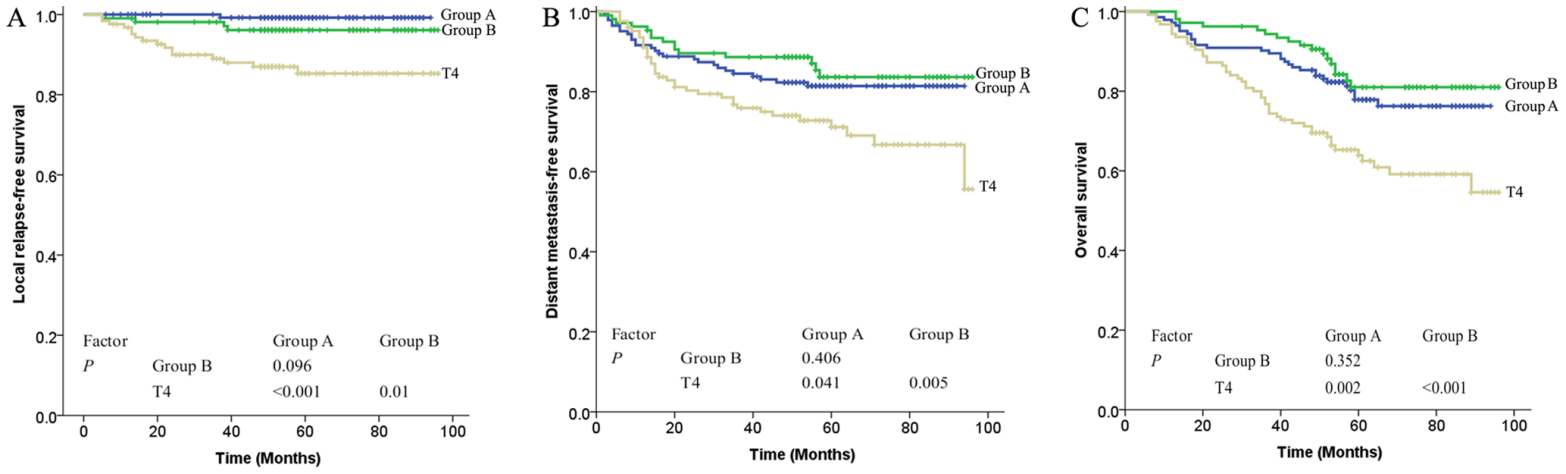
Survival rate by T-category in 375 patients: (A) local relapse-free survival, (B) distant metastasis-free survival, and (C) overall survival. Group A: T3 without MRI-detected cranial nerve involvement; Group B: T3 with MRI-detected cranial nerve involvement.

The LRFS of Stage T3 with or without MRI-detected CNI were 96.1% and 99.2%, and the LRFS for T4 patients was 87%, the DMFS were 83.6%, 81.4% and 71.1% and the OS were 81%, 77.9% and 63.9%, respectively (Figs. 4 A, B, and C). The survival (LRFS, DMFS and OS) of Stage T3 patients (with or without MRI-detected CNI) was found to be superior to that of patients with Stage T4 (P<0.05). However, there was no statistically significant difference between T3 patients with and without MRI-detected CNI (χ^2^ = 2.766, P  =  0.096; χ^2^ = 0.69, P = 0.406; χ^2^ = 0.868, P = 0.352).

**Figure 5:**
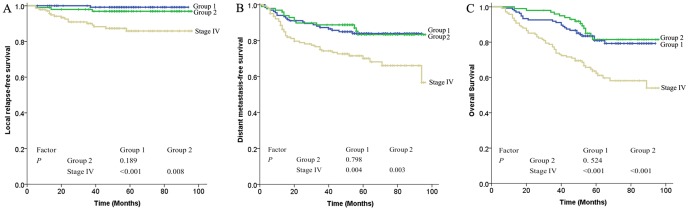
Survival rate by clinical stage in 375 patients: (A) local relapse-free survival, (B) distant metastasis-free survival, and (C) overall survival. Group 1: Stage III without MRI-detected cranial nerve involvement; Group 2: Stage III with MRI-detected cranial nerve involvement.

The survival of Stage III patients with or without MRI-detected CNI was significantly superior to that of Stage IV patients (P<0.01), whereas the difference in survival between Stage III with and Stage III without MRI-detected CNI patients was not significant for any endpoints (LRFS χ^2^ = 1.722, P = 0.189; DMFS χ^2^ = 0.065, P = 0.798; OS χ^2^ = 0.407, P = 0.524; Fig. 5A, B, and C).

Due to nearly all patients in Stage T4 (121/125, 96.8%) and Stage IV (129/141, 91.5%) having MRI-detected CNI, the comparison of survival of Stage T4 and Stage IV patients with and without MRI-detected CNI could not be performed.

## Discussion

CN palsy is common in locally advanced NPC. Although MRI has better soft tissue discrimination than CT, and has been recommended for detection of perineural involvement, the MRI findings are not always consistent with the symptoms of CN palsy [1]–[3]. The incidence of MRI-detected CNI was 60.8% (228/375) and only 18.0% (41/228) patients presented with CN palsy in the current study, which were similar to the reports from Su and Liu [2], [4]. A reasonable explanation could be that the process of CNs involvement with tumors was insidious without symptoms or the CNs are resistant to tumor invasion.

In addition, there were a total of five cases of CN palsy that we failed to discover by MRI in four patients. MRI images of these four patients were reviewed, and the failure to detect CNI may be attributed to the following reasons: i) some small branches of CN had been infiltrated by tumor but were too small to be displayed on MRI; ii) although the carotid sheath involvement can be easily detected by MRI, it is difficult to distinguish whether there is accompanying lower CN (CN IX-XII) involvement or not.

The current study revealed that >90% of Stage T4/IV patients presented with MRI-detected CNI, whereas, fewer than 43% of Stage T3/III patients had MRI-detected CNI (Table 2). The difference was statistically significant. This is because [3]: i) NPC, which originates from the pharyngobasilar fascia, can invade the cavernous sinus and middle cranial fossa through the skull base and infringe on CN II to VI. ii) NPC may also involve the carotid space invading CN XII which exits through the hypoglossal canal, and CN IX to XI which emerge from the jugular foramen (lower CN).

There are numerous available studies regarding patients with CN palsy have a poorer prognosis than those without CN palsy [8], [16]. Therefore, the 7th edition AJCC TNM staging system considered CN palsy as Stage T4, and emphasized the importance of clinical evaluation of CN palsy but not the radiology signs of CNI in staging assessments [4], [6]. Particularly, the role of radiology findings of CNI in the current staging system is still controversial [4], [17].

In the present study, the 5-year LRFS, DMFS, and OS of Stage T3 patients with or without MRI-detected CNI were similar, and were superior to those of Stage T4 patients (Fig. 4A, B, C), suggesting that Stage T3 patients with MRI-detected CN involvement should not be defined as Stage T4 and, thus, avoid excessive treatment. In addition, the survival curves of LRFS, DMFS, and OS for Stage III with or without MRI-detected CN involvement were close, but clearly separated from those for Stage IV (Fig. 5A, B, C), indicating that, regardless of MRI-detected CN involvement, Stage III was associated with a favorable prognosis.

Liu and his colleague reported that, the 3-year OS and DMFS of Stage T3 between patients with and without MRI-detected CNI were significantly different (P = 0.0087, and P = 0.0112), whereas, the difference of LRFS between Stage T3 with MRI-detected CNI and Stage T4 was not statistically significant (P = 0.0797) [4]. The inconsistency with the current study may be due to: i) conventional radiotherapy was the main treatment in that study, which may lead to a lower LRFS for T3 patients with MRI-detected CNI; ii) the OS and DMFS may be influenced by N classification, as it is generally accepted, that N classification is a primary independent prognostic factor for the OS and DMFS in the IMRT era.

The IMRT can improve local control and reduce radiation-induced toxicities by providing better tumor target coverage and is significantly better at sparing sensitive normal structures. It has been widely accepted as the standard treatment for locally advanced NPC, although it is still controversial whether early or advanced T Stages of NPC can benefit from IMRT [18], [19]. Here we demonstrate for the first time that when treated with IMRT, the survival of NPC patients in Stage T3 or Stage III with and without CNI was not significantly different, and was distinctly superior to that of Stage T4 and Stage IV. However, due to the limitations of this being a single center treatment, the results require confirmation through the accumulation of studies from more cancer centers.

Another limitation in the current study is the lack of unified chemotherapy regimens. Concurrent chemoradiation therapy has been repeatedly proven to improve survival [20], [21]. However, only 37.9% (142/375) of the patients received concurrent chemotherapy in the study. Future prospective studies of IMRT combined with concurrent chemotherapy with appropriate chemotherapy regimens and number of chemotherapeutic cycles, may be needed to verify the results.

In conclusion, the current study confirmed the high incidence of MRI detected CNI in local advanced NPC. When treated with IMRT, the LRFS, DMFS, and OS of Stage T3/III patients with or without MRI detected CNI were similar, but significantly better than patients with Stage T4/IV. Patients in Stage T3 with MRI-detected CNI should not be classified as Stage T4. This supports physical examination rather than the radiology imaging to evaluate CN involvement in the current AJCC T4 classification.
